# Getting smarter with data: understanding tensions in the use of data in assurance and improvement-oriented performance management systems to improve their implementation

**DOI:** 10.1186/s12961-018-0401-2

**Published:** 2018-12-22

**Authors:** Karen Gardner, Sue Olney, Helen Dickinson

**Affiliations:** Public Service Research Group, School of Business, UNSW Canberra, PO Box 7196, Canberra BC, 2610 Australia

**Keywords:** Quality, performance, safety, outcomes, performance indicators, performance governance, incentives, professional motivation

## Abstract

**Background:**

A better understanding of the conditions under which performance indicators can be used to improve accountability for outcomes and promote quality improvement could help policy-makers develop more effective performance management systems. One problem is the lack of conceptual models and empirical data that describe the processes through which different approaches use data together with other incentives to influence motivation.

**Discussion:**

Drawing on the performance governance and quality improvement literature, we developed a framework that distinguishes between the practice of using information to verify levels of performance in market-oriented performance management approaches and using indicators to monitor and promote improvement through building capacity for using data in service and professional networks. The framework explores how performance indicators are deployed and used in the different approaches to enact accountability or stimulate motivation for improvement and articulates the types of system architecture and processes needed to advance implementation.

**Summary:**

The framework encourages a critical appraisal of the motivations, reward systems and techniques that underpin different performance management approaches. Understanding how and for what purpose performance information is used in everyday practice will advance theory and help inform decision-makers in designing the conditions that effectively contribute to performance accountability and improvements.

## Introduction

Performance management (PM) systems are ubiquitous in healthcare and across public services more broadly. Their introduction in the late 1980s marked a major policy shift away from command and control, rule-based approaches in managing public services, to more market-based forms of governance, involving indirect and arms-length regulatory approaches that use indicators to measure performance against targets [1–3]. Performance systems may be used either to assure quality via summative information for external accountability and/or be internally driven to generate formative data for quality improvement [2]. In this way, PM approaches are both a tool of accountability as well as improvement and can be seen as a vehicle for achieving democratic ideals of making services more accountable to citizens [4]. A key assumption is that governance processes that combine greater central accountability with local empowerment can drive innovation and service improvement [5].

Typically, performance systems in health services combine the use of three key strategies to stimulate changes in practice. Specifically, they use performance indicators to highlight variations in care, mechanisms such as financial and reputational incentives to motivate behaviour change amongst professionals, and reporting/feedback processes aimed at providing consumers and purchasers (including governments) with the information they need to make more informed purchasing decisions [6]. Despite the overwhelming acceptance of PM as a policy tool in many countries [3, 4], implementation is in its infancy [7] and is not unproblematic or without contention. In primary care, PM pushes GPs and general practices into a broader managerial framework of accountability, beyond traditional modes of professional self-regulation that operated at the level of individual patient encounters, and over which the professions had a high degree of autonomy and freedom from regulation [8]. While traditional accountability mechanisms were internally driven, professionally controlled systems, new forms of PM tend to be externally driven, state-based systems using managerial rather than professional forms of regulation [9].

How best to coordinate the use of different strategies to monitor and manage progress towards achieving health goals and at the same time support constructive, collaborative, professionally driven quality improvement, remains unclear. One problem is the lack of conceptual models and empirical research that describe the processes through which different approaches use data alongside other incentives and strategies to influence motivation. This paper explores how performance indicators are deployed and information is used to promote performance in different PM systems, as well as the means by which they seek to enact accountability or stimulate motivation for improvement. To do this, we draw on the improvement and PM literature and experience in evaluating continuous quality improvement (CQI) and PM processes in primary healthcare to build a framework that distinguishes between these different goals, articulates the key processes used to support them, and the kinds of system architecture and processes needed to advance implementation. An earlier version of the framework supported conceptual development of a National CQI Framework in Aboriginal and Torres Strait Islander health services in Australia [10] and has been used in other publications [11, 12]. We identify some striking tensions and argue that, while the literature suggests that different approaches to PM are increasingly being linked through policy processes, the system architecture, key strategies and skill sets required for assurance approaches do not adequately support the widespread internal use of information for continuous improvement. Feedback loops of all kinds are underdeveloped. The paper aims to encourage a critical appraisal of the motivations, reward systems and techniques that underpin different approaches, and suggests the type of system architecture and processes needed to advance implementation in the future.

## Performance management ideal types and their contemporary examples

Burau [8] distinguishes four PM ideal types, namely markets, hierarchies, networks and self-regulation. Markets and hierarchies use managerial power and deploy strategies such as performance pay, ranking, benchmarking and competition to achieve their goals. Networks and self-regulatory systems rely on professional authority and use clinical standards, codes of practice and monitoring through peer review. Although these systems are philosophically at odds, analyses of new public management reforms in Britain [9] and more recent case studies in Germany, Denmark and New Zealand [7, 8, 13], point to the emergence of hybrid systems in which countries use a combination of strategies, but tend towards one or the other, either markets or more self-regulatory approaches, depending on individual histories and the levers available to policy to promote change [7].

**Fig. 1:**
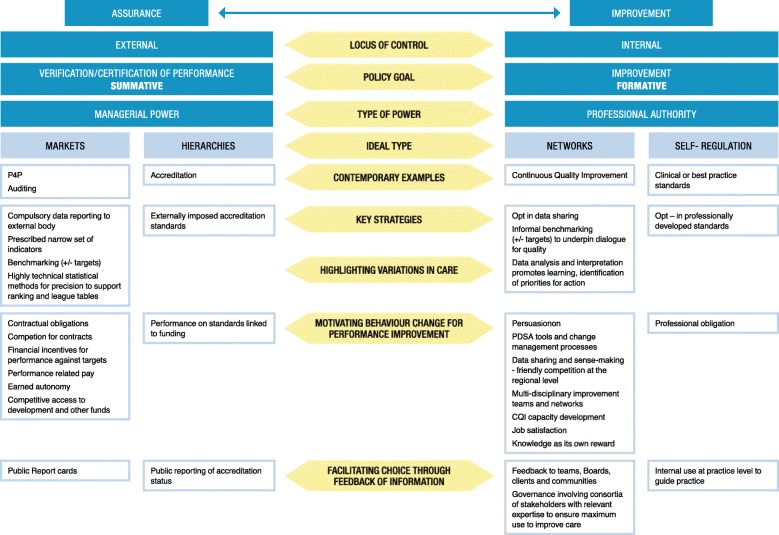
Performance management approaches and their component parts

Drawing on these four ideal types, Fig. 1 situates contemporary PM examples along a continuum, from the externally driven systems described by Freeman [2], which exercise managerial power to verify levels of performance for accountability to governments and funders (often as part of contractual funding arrangements with Ministries of Health), to more internally controlled systems based on professional authority that seek to promote quality improvement such as through Collaboratives and other structured improvement programmes. The policy goal and locus of control of each of these alternatives is identified as well as the key strategies used to underpin their implementation.

As shown, Pay-for-Performance (P4P) and auditing regimes are the key contemporary examples of market-based approaches. They seek compliance by auditing performance information, often against targets [14] or standards. The Quality and Outcomes Framework in the National Health Service England is a major contemporary example of a P4P programme. Accreditation is also an externally driven approach, but it is a key example of a hierarchical approach in which an independent external peer assessment of an organisation’s level of performance in relation to a set of standards is undertaken on a routine basis [15]. Most health services are required to achieve accreditation under funding arrangements with governments. Internally driven systems, on the other hand, are based on professional authority and use information formatively to promote improvement [2]. CQI and more traditional standards and guidelines are key examples. CQI is a structured organisational process that involves staff in planning and implementing a prospective, ongoing flow of service improvements based on continuous review of indicator data [16], usually performed as part of a Plan-Do-Study-Act (PDSA) cycle. Guidelines and standards are developed by the professions which exercise authority over specification of ‘best practice’ and their dissemination and use.

### Highlighting variations in care

As shown (Fig. 1), all four ideal types use data, measurement and benchmarking techniques to highlight variations in outcomes of care, but data are used in different ways to achieve change in practice [2]. In P4P and auditing systems, indicators are used as summative mechanisms to verify levels of performance for the purpose of achieving external accountability to funders, but in networks and self-regulatory systems data are used formatively for achieving quality improvement. Auditing and performance measurement tend to rely on ranking processes to facilitate comparison, which forms the basis of league tables that establish levels of performance so these can be linked to rewards and sanctions [2]. In the case of accreditation, data are used to assess performance against external standards and, while this is a developmental process, it is also summative – an organisation is either accredited or it is not [11].

Summative systems are usually concerned with establishing levels of performance and with whether a specific goal or target has been reached or not; they are not concerned with incremental change or improvement of individual services or cohorts per se. Similarly, assurance systems, such as accreditation, consider whether or not there is an appropriate client record or recall system in place but not the rate at which routine testing or achievement of clinical measures has occurred for clients as is the case in formative systems [11]. Data are used for certification (accreditation) and verification (auditing) and are not explicitly concerned with explaining results.

On the improvement side, CQI systems tend to use statistics to make comparisons descriptively, utilising more informal benchmarking processes as a starting point for engaging practitioners and other stakeholders in dialogue to generate insights into practice. Measurement can be less formal than in assurance systems, where the level of precision needed to compare performance and determine rank order is very high. In improvement programmes such as Collaboratives, assessment of the context in which performance is achieved is paramount for interpreting and making sense of results, and for identifying opportunities for improvement and sharing action strategies with ‘like’ services. Joint reflection enables teams to develop a more accurate picture of data and to derive meaning because it takes contextual factors into account when interpreting performance results. A primary care team, for example, might reflect on the context of non-optimal performance in relation to delivering services in accordance with recommended diabetes care guidelines, to assess whether staff shortages, patient information or some other factor/s underlie the result. When used formatively, understanding performance in context is key to learning and to identifying appropriate priorities for action.

### Motivating behaviour change

Contracting, competition for funds, financial incentives and performance-related payments are the key mechanisms used to motivate behaviour change amongst professionals in assurance systems. Some systems also reward services for reaching targets by reducing reporting requirements (earned autonomy) or offer competitive access to seed funding for ‘high performing’ services to encourage innovation [17].

In professionally based network systems, behaviour change is supported through strategies that build capacity for using data to improve practice. PDSA tools and processes that support structured deliberative dialogue are key strategies in CQI. These enable teams to use measurement and problem-solving techniques to identify unwarranted variations in care and to test and embed improvements into practice. Data sharing in multi-disciplinary teams and networks are also used to facilitate sense making, which is the active process of assigning meaning to ambiguous data, and can only occur through human reflection [18]. By supporting those who derive meaning from data to identify priorities for improving outcomes and determining actions, organizations can use data to underpin learning opportunities. Knowledge is its own reward and friendly competition motivates stakeholders.

### Facilitating choice through feedback of information

Assurance systems typically aim to provide governments, purchasers or consumers with the information they need to make more informed purchasing decisions [6]. In the case of assurance systems, public reporting of comparative data is said to help funders and consumers determine the quality of services they may wish to purchase or access. Improvement systems tend to provide data feedback internally to individual services, teams, boards, clients or communities as a stimulus for engaging in dialogue to improve practice. Dialogue is supported in structured processes such as PDSA cycles at team or service level, or communities of practice at regional levels. These enable groups to embed feedback and ongoing review into routine practice among networks of interested practitioners and other stakeholders. In this way, feedback can be used to build a dynamic approach that enhances adaptability of systems to create change.

## Tensions between assurance and improvement systems

The tension between using performance indicators for assurance or improvement purposes poses theoretical and practical challenges. At the practical level, implementation is highly context dependent and the extent to which different approaches can be implemented varies according to the pre-existing relationships and policy levers within that system. As shown in the framework, market approaches are characterised by contestability and competition for funds, where success hinges on business acumen and delivery of outcomes within set parameters, while network and professional approaches leverage on actors’ legitimacy and authority to build relationships across boundaries to improve and/or redefine desired outcomes.

In Australian primary care, implementing auditing or P4P for specific indicators and targets has proven very difficult, not least because GPs are predominantly small businesses and do not have a direct contractual relationship with government but also because there is no nationally computerised clinical information system and administrative data, which is based on fee claims, is inadequate for measuring outcomes [19]. In the absence of a contractual vehicle, the key government strategy since the late 1990s has revolved around supporting the development of a professional structure (once Divisions of General Practice through various iterations to what are now Primary Health Networks) through which relationships are built with the profession to engage them in policy development and best practice primary care approaches [20]. Notwithstanding the successes or otherwise of that approach, GPs have thus far resisted more recent market-oriented attempts to impose a PM system that requires them to draw on data from their own practices and report it directly to government, but have taken up formal improvement approaches over time [21]. Financial incentives have been paid to individual practices to encourage (reward) them to adhere to best practice treatment guidelines for certain conditions, such as diabetes, and more recently to engage in using and sharing practice data for structured quality improvement purposes [21]. Indigenous primary healthcare services, on the other hand, are directly funded by the Australian government and contractually required to report on specific indicators, drawn from practice level data that are aggregated and routinely publicly reported [11, 22]. While reporting is a requirement for funding, there has been concern in that sector that current comparisons of performance are high level and, while they may be useful for sector level discussions, they are inadequate for stimulating improvement practices at a local level as feedback is too infrequent and comparisons may not be with peer services [11].

The framework also highlights the different types of system architecture required to operationalize different approaches. Although modern PM systems of all types are increasingly reliant on web-based portals that enable data extraction, transfer and sharing capabilities, feedback loops and timeframes for reporting differ significantly between approaches. CQI programmes use data prospectively within teams and networks and therefore require system architecture that can support data flows and feedback loops over which providers have control at different levels of the system, or in different jurisdictions, or over different timeframes (services, regions, state, etc.) and with greater frequency to enable ‘real time’ feedback of results, tracking of individual performance and with in-built capacity for comparing de-identified results among services. Such approaches recognize a diversity of actors involved, as well as a diverse range of processes and subsystems in which feedback loops are required [5]. Assurance systems feed back information more infrequently and often only to the general public in the form of league tables or comparative performance, rather than to services, which may need to run their own analyses and share data within their chosen networks.

A fundamental difference between such systems is that network approaches are learning systems that motivate improvement through engagement, and the required system architecture enhances adaptive capacity for change. Assurance systems are static and linear, require one way and more infrequent feedback, revolve around financial rewards and sanctions, and are implemented through competition for contracts. Although proponents of assurance systems frequently argue that a policy focus on improvement is intended, the architecture or transfer of control necessary to enact it is rarely implemented and reporting too often becomes little more than a tick-box exercise for those involved. A vast literature testifies to the wealth of unintended consequences and to the propensity of such systems to undermine the very conditions required for collaboration [23, 24]. Recent evidence from systematic reviews [25, 26], large evaluations and studies of major health programmes in the USA and UK point to their failure to demonstrate improvements in health outcomes [27] or care continuity [28]. The expense of implementation and whether this justifies the outcomes achieved has been debated [29] and empirical work suggests that, rather than embedding improvements in healthcare, financial incentives are associated with a decline in activity when withdrawn [30] and may undermine professional motivations and trust [31]. Network systems, on the other hand, are complex and hard to implement as they require multiple actors, ongoing commitment, leadership and time. Literature suggests that their effectiveness also depends on the inclusion of key stakeholders, participant willingness to pool or shift resources and their capacity to develop common conceptions of problems, to find balance between inconsistent goals, and to develop innovative solutions [32–36]. Yet, organisations tightly bound by regulations, contractual obligations, performance indicators, eligibility criteria and incentives tied to the key performance indicators of their funding source have little scope to work that way and are generally reluctant to stray beyond the boundaries of their ‘measurable activity’ in a contestable funding environment [37].

As implementation of hybrid approaches to PM advances in different countries [7], a more complex picture of areas amenable and not amenable to different incentives and strategies emerges [27]. It is hoped this framework will promote a deeper understanding of the inherent tensions between PM approaches and the practical challenges associated with their implementation. Further thinking is needed on how, when and in what circumstances different mechanisms might be used to build a coherent system within the constraints of context. Coherent systems need to move beyond alignment of different strategies to looking at supporting how data can be used to motivate stakeholders in collective efforts to achieve ‘good’ outcomes. At times, paying for improvements or supporting the introduction of new practices may be warranted [38–40] and frameworks currently exist to assist policy-makers determine when to use financial incentives or another approach [41].

Without understanding the different PM goals, strategies and the system architecture that shape the use of data for achieving accountability and improvement, or acknowledging the system constraints that dictate possibilities for implementing performance indicators and related incentives, the policy focus on incentivising behaviour change is likely to become bogged down in compliance and accountability processes, resulting in rigid systems with limited capacity for innovation and adaptation. Building an integrated system must recognise that quality is a collaborative rather than an individual effort, that the health landscape is not a level playing field, and that financial rewards play a small part in an overall system that uses multiple incentives and builds relationships to achieve improvement. Less focus on sticks than carrots and more on collaboration than on competition in achieving results is required, as are supportive infrastructure and tools.

## Conclusion

A coherent approach to PM will remain elusive as long as considerations on exactly how data can be used to achieve different goals remains poorly articulated. The framework can be used by policy-makers, practitioners and researchers as a conceptual map to identify the key approaches to performance management and the strategies they deploy for using data to promote improvement and accountability to stakeholders, including governments. Applying the framework as a research aid in different settings may enhance our understanding of the system architecture, processes and mechanisms required to enhance motivation for change and the kinds of intended and unintended consequences that may arise. Understanding how and for what purpose performance information is used in everyday practice will advance theory and help to inform decision-makers in designing the conditions that effectively contribute to performance and accountability improvements [42].
